# Point-to-point associations of drusen and hyperreflective foci volumes with retinal sensitivity in non-exudative age-related macular degeneration

**DOI:** 10.1038/s41433-023-02554-4

**Published:** 2023-05-11

**Authors:** Gregor S. Reiter, Hrvoje Bogunovic, Ferdinand Schlanitz, Wolf-Dieter Vogl, Philipp Seeböck, Dariga Ramazanova, Ursula Schmidt-Erfurth

**Affiliations:** 1 Laboratory for Ophthalmic Image Analysis, Department of Ophthalmology and Optometry, Medical University of Vienna, Vienna, Austria; 2 Vienna Clinical Trial Center (VTC), Department of Ophthalmology and Optometry, Medical University of Vienna, Vienna, Austria; 3 RetInSight GmbH, Vienna, Austria; 4 Center for Medical Statistics, Informatics and Intelligent Systems, Medical University of Vienna, Vienna, Austria

**Keywords:** Retinal diseases, Prognostic markers

## Abstract

**Objectives:**

To evaluate the quantitative impact of drusen and hyperreflective foci (HRF) volumes on mesopic retinal sensitivity in non-exudative age-related macular degeneration (AMD).

**Methods:**

In a standardized follow-up scheme of every three months, retinal sensitivity of patients with early or intermediate AMD was assessed by microperimetry using a custom pattern of 45 stimuli (Nidek MP-3, Gamagori, Japan). Eyes were consecutively scanned using Spectralis SD-OCT (20° × 20°, 1024 × 97 × 496). Fundus photographs obtained by the MP-3 allowed to map the stimuli locations onto the corresponding OCT scans. The volume and mean thickness of drusen and HRF within a circle of 240 µm centred at each stimulus point was determined using automated AI-based image segmentation algorithms.

**Results:**

8055 individual stimuli from 179 visits from 51 eyes of 35 consecutive patients were matched with the respective OCT images in a point-to-point manner. The patients mean age was 76.85 ± 6.6 years. Mean retinal sensitivity at baseline was 25.7 dB. 73.47% of all MP-spots covered drusen area and 2.02% of MP-spots covered HRF. A negative association between retinal sensitivity and the volume of underlying drusen (*p* < 0.001, Estimate −0.991 db/µm^3^) and HRF volume (*p* = 0.002, Estimate -5.230 db/µm^3^) was found. During observation time, no eye showed conversion to advanced AMD.

**Conclusion:**

A direct correlation between drusen and lower sensitivity of the overlying photoreceptors can be observed. For HRF, a small but significant correlation was shown, which is compromised by their small size. Biomarker quantification using AI-methods allows to determine the impact of sub-clinical features in the progression of AMD.

## Introduction

Age-related macular degeneration (AMD) remains the major cause of irreversible blindness in the developed countries. Named the ‘priority eye disease’ by the World Health Organization, it has a significant impact on mental health and quality of life of affected patients [1]. In the early stage of this chronic disease, characterized by the accumulation of drusen and focal pigmentary alterations, such as hyperreflective foci (HRF), within retinal layers, visual acuity can remain stable for a long time, although patients often describe subtle alterations, such as deteriorating of vision in dim light situations or at night [2]. These alterations have been associated with decreased sensitivity of the rod system responsible for scotopic vision and delayed dark adaptation [3].

Whereas significant improvement in the therapy of exudative AMD was made, treatment of non-exudative AMD remains limited [4]. To support the development of treatment for early AMD, several studies tried to identify functional endpoints that may help to expose pathophysiological processes before visual loss occurs in the advanced stages of disease. These studies involved tests such as dark adaptation time, low luminance visual acuity, cone-mediated flicker sensitivity and microperimetry [5–7]. Histologic and optical coherence tomography (OCT) studies depict how drusenoid accumulations lead to a compression of overlying photoreceptors [8], however, a direct impact on reduced sensitivity remained controversial. Studies using microperimetry found a significant relationship between a decreased macular sensitivity and large drusen volumes [9, 10].

This study investigates the impact of individual drusen and HRF in early and intermediate AMD on retinal sensitivity, in order to determine whether the size and location of drusen or HRF themselves, rather than generalized pathophysiological processes throughout the retinal layers including Bruch’s membrane in early and intermediate AMD are responsible for the decrease of the functionality of the photoreceptors. To achieve this goal, an artificial-intelligence-supported point-to-point analysis was performed where the regional measurements from the automated segmentation of drusen and HRF using spectral-domain OCT are compared to the corresponding microperimetry measurement points.

## Methods

All examinations were performed at the Medical University of Vienna. The study protocol was approved by the local ethics committee and adhered to the Declaration of Helsinki. The examinations listed below were included in this study protocol, and the patients had to give their written informed consent before inclusion to the study.

### Inclusion/exclusion criteria

Consecutive patients with drusen in early and intermediate AMD were recruited from the outpatient clinic of our department. An experienced retinal specialist examined the patients and classified eyes according to the Beckman classification [11]. Patients presenting signs of advanced AMD, e.g. atrophic retinal areas or neovascularization were excluded, as well as eyes presenting other eye diseases than AMD, as well as patients with previous surgery other than uncomplicated cataract surgery.

### Imaging procedures

Patients meeting the protocol criteria were informed about the study aims and procedures. After giving written informed consent, patients were included in the study and underwent a standardized examination. At every visit, best corrected visual acuity (BCVA) was obtained first and mydriatic eye drops were administered thereafter.

**Fig. 1:**
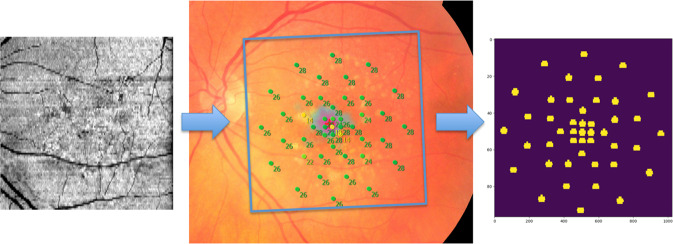
The pseudo-SLO image of SD-OCT scans (left) was registered to the colour fundus image of the MP-3 device (middle). The exact location of each MP-spot was then transferred to the corresponding OCT A-scans (right, in yellow).

Microperimetry assessment was conducted in mesopic conditions after 5 minutes of dark adaptation, with the fellow eye patched. Using the Microperimeter-3 (MP-3, Nidek, Gamagori, Japan) high-definition colour images of the fundus overlapping the microperimetry stimuli were acquired. The customized high-density pattern of the microperimetry examination consisted of 45 stimulus test locations in 8° around the fovea with increased density at the fovea (Fig. 1). The fixation target was shown as the 1° red circle, centred at the fovea. The MP-3 measurement was carried out by a 4-2-1 staircase strategy with the Goldmann III stimulus size. Maximum luminance of MP-3 was 10,000 asb, and the stimulus dynamic range is between 0 and 34 dB. The automatic fundus eye tracking system enables accurate projection of stimulus in order to ensure the same points on retina during the follow-ups. Subsequently, the macular area was scanned using the Spectralis SD-OCT system (Heidelberg Engineering, Heidelberg, Germany), with a pattern of 97 B-scans (1024 A-scans) covering an 20 × 20° area, eye tracking and follow-up mode enabled. Follow-up examinations were acquired in three-monthly intervals over 12 months. Therefore, up to 5 examinations can be available per eye during the 12 months observational period.

### OCT image segmentation

**Fig. 2:**
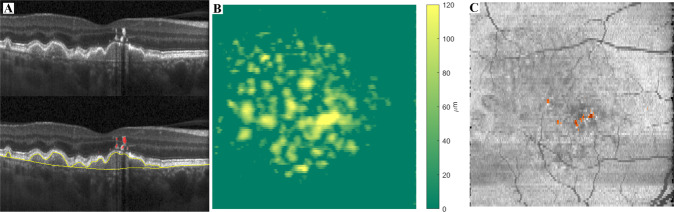
Examples of the automatic segmentation of drusen and hyperreflective foci (HRF). **A** OCT b-scan with drusen between Bruch’s membrane and the inner border of the retinal pigment epithelium (between yellow lines) and HRF (red). **B** Enface drusen map, colour coded for drusen thickness. **C** Enface map of HRF overlaying the pseudo-SLO image.

OCT scans were processed with fully automated image segmentation methods. Intraretinal layers and Bruch’s membrane (BM) were segmented with Iowa Reference Algorithms [12, 13]. En-face drusen thickness maps were then obtained as the distance between the outer retinal pigment epithelium (RPE) and BM surfaces. HRF were automatically segmented with a deep learning approach based on a convolutional neural network (CNN) [14]. Volumes of drusen and HRF within a local region were calculated by adding the A-scan thicknesses within the region and multiplying with the unit OCT pixel area (0.000362 mm^2^). An example of the drusen and HRF quantification and the enface outputs are shown in Fig. 2.

### Microperimetry and OCT alignment

To obtain correspondence between microperimetry sensitivity measures and SD-OCT topology, we had to transform the exact microperimetry positions as provided by the MP-3 tool to positions in the corresponding OCT provided by Heidelberg Spectralis. For that reason, we registered the MP-3 colour fundus photograph (CFP) with the scanning laser ophthalmoscopy (SLO) image that was acquired simultaneously with the SD-OCT image. SLO and OCT images are already aligned by the scanner software. As the MP-3 scanner software does only provide the measurement locations in degree units with respect to fovea centre, but withholding the exact position of fovea centre position on the CFP, we furthermore manually defined this position and the scaling of the microperimetry directly on the CFP. To be able to precisely position the grid, we used a by scanner software annotated version of the CFP, where microperimetry measurement points and fovea centre position are overlayed on the CFP (Fig. 1).

**Fig. 3:**
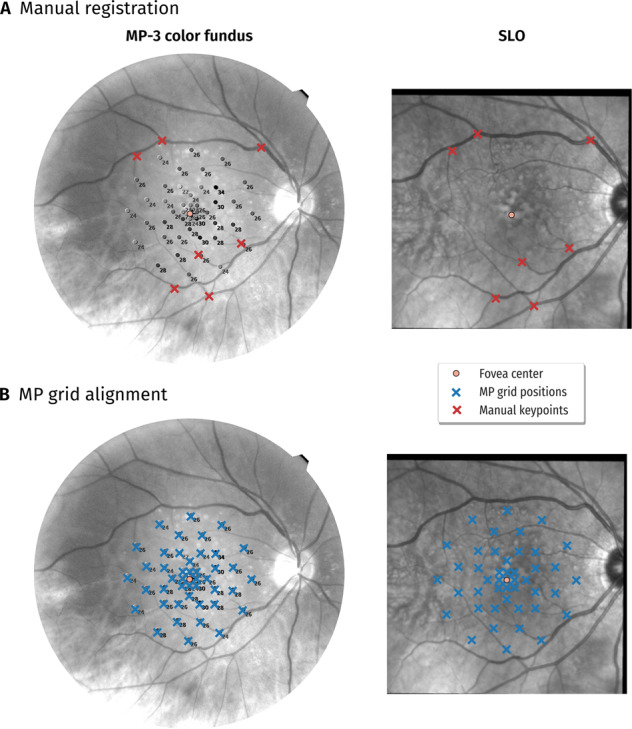
Example of manual alignment of MP-3 colour fundus photograph (CFP – black/white for better contrast) with scanning laser ophthalmoscopy (SLO) images and microperimetry measurement grid with CFP. **A** A user selected keypoints in the CFP and at the same anatomic location in the SLO (red crosses) in order to align CFP with SLO. **B** Fovea centre position and scaling of grid were manually set such that MP measurement locations match with CFP (left). Final locations are presented on SLO to the user for validation (right).

We used an in-house developed program to perform the task of CFP-to-SLO registration and pattern alignment. In terms of image registration, a user marked for each eye at least 5 keypoints on the CFP, preferable at distinguishable locations, such as vessel branches. The same keypoints were then marked on the SLO image (Fig. 3A). Having the list of corresponding keypoint positions, least-squares was used to estimate an affine transformation. With that transformation any point on CFP can be mapped to SLO. In order to align the MP-3 measurement grid with CFP, the user marked the fovea centre position in the annotated CFP and scaled the size of the pattern such that it matched the one overlayed on CFP (Fig. 3B). Finally, the transformed pattern positions on the SLO image were presented to the user for visual inspection of the correct placement of the sensitivity point positions. The Microperimetry tool provide point coordinates of measuring locations in the retina. We transformed these MP point coordinates to coordinates in the OCT image via image registration. As the region of interest (or blob) per transformed measuring point was determined directly in the OCT image, the size of the region of interest did not vary between the measuring points.

### Statistical analysis

Nested mixed models were analysed for retinal sensitivity depending on the fixed effects drusen volume, HRF volume and visit as covariate due to a well-known learning effect of microperimetry testing, allowing also for interaction between visit and drusen volume, and nested random effects of MP-spot within eye and eye within patient. Eccentricity (in degree distance from the foveal centre point) and the interaction of drusen volume with eccentricity were further included in the model. All descriptive and statistical analyses were conducted using R (version 4.1.3).

## Results

In total, 8055 point-to-point matched stimulus points from 179 visits from 51 eyes of 35 consecutive patients with drusen secondary to early and intermediate AMD in at least one eye were included in this prospective study. The mean age of the patients was 77.4 ± 6.6 years (range 60.3–85.7), 18 (51%) were female. The median drusen volume per MP-spot at baseline was 0.01 × 10^−3^ mm^3^ (IQR 0–0.08 × 10^−3^; range 0–0.016), mean retinal sensitivity at baseline 25.71 ± 3.53 dB (range 0–34).

At baseline, 73.47% of all MP-spots of all eyes had a drusen volume > 0, with a median volume of 0.03 × 10^−3^ mm^3^ per MP-spot (IQR 0.07 × 10^−3^–1.14 × 10^−3^; range 0–0.016). The presence of HRF in the comparatively small, defined MP-Spots was 2.02%, with a median volume of 8.46×10^−5^ mm^3^ (IQR 6.74 × 10^−5^–11.42 × 10^−5^; range 6.09 × 10^−5^–30.59 × 10^−5^). In order to rule out eventual segmentation errors, only HRF volumes smaller than 6 × 10^−5^ mm^3^ were set 0.

Calculating a simplified mixed model based on the presence of drusen and HRF (without consideration of their respective volume), we found decreased retinal sensitivity of −1.297 db (*p* < 0.001) for drusen presence and −1.422 db (*p* < 0.001) for HRF presence.

Drusen volume was significantly associated with reduced retinal sensitivity (Estimate: −0.991 db/µm^3^; 95% CI: −0.887 to −1.095; *p* < 0.001). Likewise, HRF volume was also significantly associated with reduced retinal sensitivity (Estimate −5.230 db/µm^3^; 95% CI: −1.907 to −8.559; *p* = 0.002). Due to the multivariate structure of our model, both drusen volume and HRF volume have a significant impact on retinal sensitivity while correcting for each other’s value. The estimates of reduced sensitivity can be interpreted as a decrease of −0.991 db per cubic millimetre drusen volume and −5.230 db per cubic millimetre HRF volume in each individual microperimetry stimulus, respectively.

**Fig. 4:**
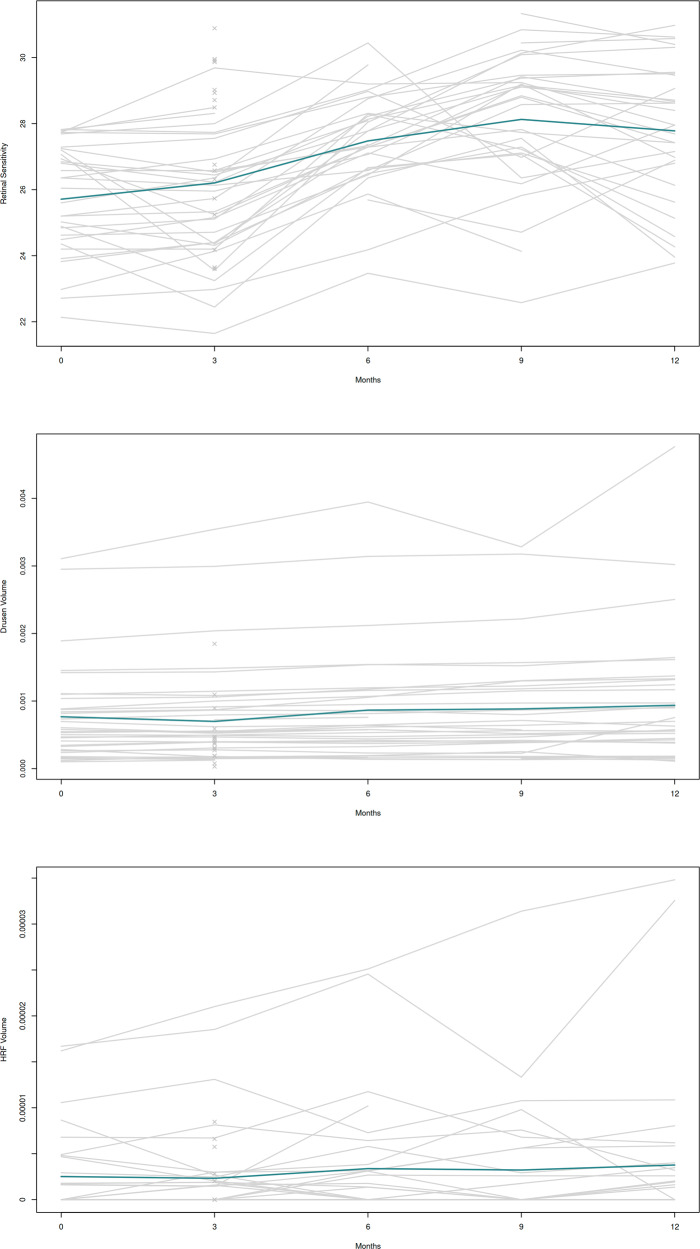
Individual mean measurements during the 12-month follow-up. Retinal Sensitivity (top), Drusen volume (centre) and HRF volume (bottom). Each grey line represents the mean respective value in each eye of each patient. Each light grey cross is the mean value in each eye with only one measurement. Bold blue line connects the means at each measurement point.

Overall retinal sensitivity increased by a mean of 0.242 db/visit (95% CI: 0.23 to 0.25; *p* < 0.001), which is consistent with a learning process already known [15]. Besides retinal sensitivity, drusen volume grows over time, as does HRF volume (see Fig. 4). The factor “visit” was included in the multivariable model and the above mentioned estimates of HRF and drusen volumes were corrected for this learning effect. A significant interaction of drusen volume with visit (*p* < 0.001) was also included in the statistical model which also resulted in a better model performance based on the information criterion (AIC).

To further investigate the learning effect, we started with the original model which resulted in an increase of 0.242db/visit. In a second step, we recalculated the model including all fixed and random factors described in the methods section, but excluded the baseline visit. The estimate for change over time did not change much from its original value (0.258 db/visit), but remained significant in this second model (*p* < 0.001). In a third step, we also excluded the second visit and recalculated the model with the last three visits available. The estimate for change over time decreased to 0.046 db/visit, but still remained significant in the model (*p* < 0.001).

The factor eccentricity was slightly positive (0.086 db/degree, 95% CI: 0.05 to 0.12, *p* < 0.001) and the interaction of drusen volume with eccentricity was also significant (*p* < 0.001) and improved model performance based on AIC. This indicates a small increase of retinal sensitivity with increased eccentricity, presumably due to a decrease in retinal sensitivity in the foveal area based on the higher drusen load, which was however considered by including the interaction of drusen volume and eccentricity. This was confirmed by a subsequent experiment matching MP stimuli to their ETDRS sections and using the ETRDS sections instead of eccentricity. In this experiment retinal sensitivity was significantly decreased in sector 1 (foveal 1 mm) by a mean of 1.689 db compared to the other sectors. The only significant pairwise post-hoc differences were identified between section 1 with all other ETDRS sectors (all *p* < 0.001). After correcting for multiple testing using Bonferroni correction, no further differences between sectors were found.

During follow-up, no eye developed any form of advanced AMD (either geographic atrophy or macular neovascularization).

## Discussion

In this study, the direct impact of drusen volume and hyperreflective foci on mesopic retinal sensitivity of the overlying photoreceptors was investigated. This was supported by calculating AI-based point-to-point correlation using a set of algorithms that on one hand can quantify structural OCT biomarkers, and on the other, can place in precise spatial correspondence the microperimetry measurements and the OCT scans. As a result, a direct relationship between drusen and HRF volume, and a deteriorating retinal sensitivity of the affected photoreceptors was revealed.

Earlier studies could show that macular sensitivity measured by microperimetry is reduced in patients with early and intermediate AMD, compared to age-matched control groups [16]. In order to determine the causes, recent investigations correlated macular morphology and microperimetry, using OCT to identify and quantify anatomic lesions. As a result, morphologic characteristics that indicate degeneration of the photoreceptors, such as disruptions in the ellipsoid zone or RPE atrophy, cause a significant loss of retinal sensitivity. However, for drusen, discussion remained somewhat controversial. Whereas some surveys showed no correlation between drusen volume and retinal sensitivity, tested under mesopic conditions [17, 18], others found a negative association between retinal sensitivity and the overall drusen volume, using mesopic as well as dark-adapted microperimetry [9, 10, 19].

Drusen exhibiting a progression consisting of growing and collapsing [20] hinders determining its impact on retinal sensitivity, as the site of a collapsed druse might well preserve its low photoreceptor functionality. Furthermore, one study found a decline of sensitivity prior to development of CNV [21]. However, within 12 months of observation time of our study, no eye showed signs of significant drusen collapse or developed advanced forms of AMD. Therefore, the question whether a deterioration of retinal sensitivity in the advent of CNV or atrophic events might happen as a sign of photoreceptor cell death before it is visible with imaging methods, still remains.

Previous investigations focused on retinal layer morphology to determine associations with retinal sensitivity. Foremost, a higher thickness in the RPE-drusen complex was correlated with decreased retinal sensitivity [22, 23]. Further, decreased thickness of the outer nuclear layer (ONL) and photoreceptor thickness was also associated with reduced sensitivity [24]. Notably, foveal measurements of microperimetry were not considered in all studies [24], although cone photoreceptors are mainly distributed in the fovea and so is drusen volume and drusen growth [25, 26]. We therefore used a high density microperimetry pattern with special focus on the foveal area to investigate retinal sensitivity.

Photoreceptor integrity and retinal sensitivity assessed by microperimetry are closely linked and can be assessed using the combination of adaptive optics OCT and microperimetry [27, 28]. Since the RPE is strongly involved in the nourishment of photoreceptors, it becomes apparent that increased degeneration of the RPE might ultimately result in reduced photoreceptor function [29]. Progressive degeneration of the RPE has been described in previous investigations and increases with disease progression in non-exudative AMD [30, 31]. Hyperreflective foci are a subclinical feature of disease progression and can particularly be found in areas where atrophy of the RPE will develop [32, 33]. Direct, point-wise correlations between retinal sensitivity and HRF have not been specifically investigated so far. HRF were considered only as a generalized morphologic sign of non-exudative AMD. At that time, only the correlation of the presence of HRF [34], or the number of HRF within predefined large sectors (e.g. ETDRS sectors) of the macula, and not point-wise, was performed [22]. However, there was still a significant impact of HRF on retinal sensitivity, making the further exploration of this feature worthwhile. Later, Roh et al. found no association between the presence of HRF within an ETDRS sector and retinal sensitivity in a multivariable analysis [18]. The volume of HRF compared to retinal tissue in AMD is small. An averaging over large areas will therefore result in a diluted parameter. Compared to these previous approaches, our investigation used AI to pinpoint the exact location of each HRF and allows for point-to-point correlation with the quantitative volume of HRF and not only their presence. HRF is frequently present above large drusen or drusenoid pigment epithelium detachments. Even with the consideration of drusen volume in the same model, we found a highly significant negative association between HRF volume and retinal sensitivity. This highlights the importance to precisely identify, localize and quantify retinal biomarkers for structure/function correlations.

Reduced sensitivity indicated by higher HRF volume might therefore be due to progressive degeneration of the RPE when a HRF segregates from the RPE and migrate into the inner retinal layers [33]. Considering the migration depth of the foci did not reveal any impact on retinal sensitivity in previous investigations [22]. Thus, the impact of HRF might be present when the foci segregates from the RPE or earlier as a sign of local RPE degeneration. From there, the dysfunctional RPE leads to degeneration of rods and later cones [8], which thereafter results in the reduced mesopic microperimetry testing seen in our investigation.

The follow up of one year did not allow for the analysis of regression of drusen load which is consistent with previous studies investigating morphological changes during AMD progression [35]. Therefore, a limitation of this study is the short follow up time, where drusen regression and conversion towards advanced AMD were unlikely. Longer follow up might reveal specific patterns for the conversion towards geographic atrophy or exudative AMD. However, including short-term follow up allowed for correction of the known learning curve for microperimetry testing. Further, we did not include a control group in this study since the aim was to investigate point-to-point associations with drusen and HRF and a healthy cohort would not present with these features. The strength of our investigation is the high density, 45 spot microperimetry pattern, which covers the macular area in a comprehensive manner. In addition, we corrected for the learning effect by including multiple visits per patients, as well as the appropriate nested random effect, into our statistical model.

In conclusion, larger drusen volume was associated with reduced mesopic retinal sensitivity in our AI-supported point-to-point quantitative analysis using a high-density microperimetry assessment. The same holds true for HRF volumes in the point-to-point analysis. Increased drusen load and increasing HRF volume might indicate progressive degeneration of the RPE with already existing decrease of cone function. Point-wise connection of functional microperimetry testing and structural OCT imaging gives insight into continuous degenerative processes in AMD and their pathway to irreversible loss of visual function. Sensitive and easy to acquire progression markers are desperately needed to monitor the vast number of patients with AMD and to manage this yet unstoppable disease in its early stage with future treatments.

## Summary

### What was known before

- Visual function and retinal sensitivity is often preserved in early age-related macular degeneration.
- Functional assessments are time-consuming and prone to subjective error.
- Using retinal morphology to deduce function might improve evaluation of early disease progression.

### What this study adds

- AI-supported mapping of microperimetry and optical coherence tomography reveals novel insight into early disease progression.
- Point-to-point overlap of MP and OCT identified drusen and HRF volumes to be negatively associated with retinal sensitivity.

## Data Availability

Correspondence and requests for materials should be addressed to US-E.
